# Influence of Extrusion Parameters on the Mechanical Properties of Slow Crystallizing Carbon Fiber-Reinforced PAEK in Large Format Additive Manufacturing

**DOI:** 10.3390/polym16162364

**Published:** 2024-08-21

**Authors:** Patrick Consul, Matthias Feuchtgruber, Bernhard Bauer, Klaus Drechsler

**Affiliations:** Chair of Carbon Composites, Department of Aerospace and Geodesy, Technical University of Munich, 85748 Garching, Germany; matthias.feuchtgruber@tum.de (M.F.); bernhard.georg.bauer@tum.de (B.B.); klaus.drechsler@tum.de (K.D.)

**Keywords:** material extrusion, additive manufacturing, high-performance polymers, PAEK

## Abstract

Additive Manufacturing (AM) enables the automated production of complex geometries with low waste and lead time, notably through Material Extrusion (MEX). This study explores Large Format Additive Manufacturing (LFAM) with carbon fiber-reinforced polyaryletherketones (PAEK), particularly a slow crystallizing grade by Victrex. The research investigates how extrusion parameters affect the mechanical properties of the printed parts. Key parameters include line width, layer height, layer time, and extrusion temperature, analyzed through a series of controlled experiments. Thermal history during printing, including cooling rates and substrate temperatures, was monitored using thermocouples and infrared cameras. The crystallization behavior of PAEK was replicated in a Differential Scanning Calorimetry (DSC) setup. Mechanical properties were evaluated using three-point bending tests to analyze the impact of thermal conditions at the deposition interface on interlayer bonding and overall part strength. The study suggests aggregated metrics, enthalpy deposition rate and shear rate under the nozzle, that should be maximized to enhance mechanical performance. The findings show that the common practice of setting fixed layer times falls short of ensuring repeatable part quality.

## 1. Introduction

AM is a family of production techniques that enables the automated production of complex geometries with low material waste and lead time without needing tooling [1]. Among the AM technologies, MEX is the most common [2]. In desktop-sized printers, it is known as Fused Filament Fabrication (FFF) or Fused Deposition Modeling, but it can also be scaled to large formats. These machines often use single screw extruders for material plasticization, and the process is referred to as LFAM or Extrusion Deposition Additive Manufacturing (EDAM) [3,4].

MEX has thus far primarily been used for prototyping and to produce tooling and molds in more advanced applications, especially as LFAM. However, using technical or high-performance polymers with fiber reinforcement, the technology can also address the production of end-use parts replacing both metals and other manufacturing techniques. The granulate fed single screw extruders of LFAM systems are capable of processing materials with high fiber content from a cost-effective feedstock and at high material output. This makes LFAM both technologically and economically attractive.

The implementation of AM by MEX, both on small and large scales, is challenging due to the quality of mechanical properties in the produced material. MEX deposits material along lines in stacked layers to form the part. The processing conditions of each line, as well as the conditions of the layer being built upon, affect how well these bond together and can significantly affect the quality of the part [5,6,7]. Therefore, a comprehensive understanding of the interactions between the process parameters is the foundation for optimizing the quality towards the requirements of end-use parts.

Material selection for MEX often has relied on polymers originally developed for injection molding, which had been adapted for AM using additives and fillers during the compounding process. This generally results in a compromise between the needed properties in the final part and the processability. Adding fiber reinforcement, particularly carbon fibers, offers benefits both to the final part and processing. It improves the material’s strength and stiffness and, particularly relevant to molds, limits the coefficient of thermal expansion (CTE). The low CTE reduces warping and stabilizes the part at higher temperatures during processing [8].

Initially, the polymers used in MEX were predominantly amorphous grades, as crystallization hinders the bonding of the printed lines and layers in the printing process. Crystal formation stops chain mobility and, thus, bonding of the layers by diffusion across the interface. Crystallization introduces a time dependence of layer bondability in addition to the temperature dependence and elevates the bondable temperature. Most materials developed for injection or compression molding have been optimized to accelerate crystallization and reduce the cycle time in these processes, resulting in inferior performance in MEX, particularly in LFAM [9].

In recent years, new grades of semi-crystalline polymers have been specifically developed for AM. These materials possess tailored crystallization kinetics, with some of these materials crystallizing slow enough to be printed fully amorphous [9]. This enables the deposition of lines on amorphous layers, followed by gradual crystallization as the following layers are built, thereby showing amorphous behavior during the critical stages of processing, but then developing crystallinity and the associated favorable properties, while avoiding additional annealing steps in post-processing [10].

Polyaryletherketones (PAEKs), such as Polyetheretherketone (PEEK) and Polyetherketoneketone (PEKK), have particularly benefited from these developments, with several manufacturers introducing new grades. These polymers are used in industries striving for the highest performance, like aerospace, oil and gas, and medical, which have already been early adopters of AM as an innovative technology. PAEK materials, while costly, are valued by these industries for their superior thermal stability, chemical resistance, and mechanical properties, making them suitable for high-performance applications [11,12,13].

The objective of this study is to investigate the effect of extrusion parameters on the mechanical properties of the final parts in LFAM using a slow crystallizing, short carbon fiber-reinforced PAEK material. A set of experiments is conducted varying the line width, layer height, layer time, and extrusion temperatures [14]. The resulting layer contact temperature is measured during processing. We attempted to determine fiber misalignment in the lines after printing from cross-section microscopy, and crystallization is reproduced by replicating the thermal history in differential scanning calorimetry (DSC) measurements. Through this intermediate step of dynamic in-process temperatures and material microstructure, the observed effects on material strength and stiffness are expected to be better explained. The printed parts are evaluated by three-point bending to assess the mechanical properties along the extrusion lines and between the layers. The understanding of the impact of basic extrusion parameters and their interactions on the resulting material quality, including macroscopic mechanics and microstructural features like crystallinity and fiber alignment, can then be used to optimize processes for future high-performing applications for AM, transitioning further from the original use in prototyping towards direct part manufacturing.

## 2. Materials and Methods

### 2.1. Materials

**Figure 1:**
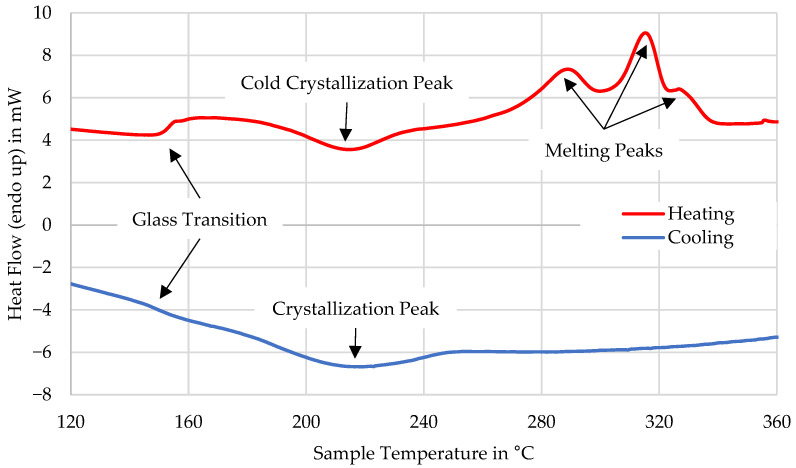
DSC measurement at 10 K/min.

The material used is a 30 wt.% short carbon fiber-reinforced PAEK developed for AM by Victrex plc (Thornton Cleveleys, UK). The recently commercialized AM200 stems from the same approach and is comparable, yet this material has an even slower crystallization behavior. DSC measurements showed that for constant cooling rates above 20 K/min, the crystallization is completely suppressed. The onset of cold crystallization is at 193 °C and it has three melting peaks at 288 °C, 316 °C, and 328 °C, and the glass transition is at 154 °C. An exemplary DSC curve is shown in Figure 1. The material’s base polymer has previously been investigated and has shown significant potential for LFAM with a 650% improvement compared to an injection molding grade [15]. The material was compounded and provided directly by Victrex.

### 2.2. Sample Preparation

#### 2.2.1. Large Format Additive Manufacturing

The material was dried for at least eight hours at 120 °C in a Vismec (Padua, Italy) DP80 dryer and fed directly to the single screw extruder by an air conveyor. An AM Flexbot by CEAD (Delft, The Netherlands) with a G25 extrusion unit, consisting of a single screw extruder with 25 mm screw diameter and a 5 cc volumetric gear pump for accurate material dispensing, was used to manufacture the samples. An 8 mm brass nozzle was used for printing. The heat zones were set to 330 °C and 350 °C for the first two, with the remaining three zones (extruder zone 3, gear pump, and nozzle) set to the values of the design of experiments. The print bed was heated to 100 °C and the machine was enclosed in a protective cell which maintained an average ambient temperature of 31 °C (minimum 25 °C, maximum 33 °C) during printing; the ambient temperature for each print was recorded.

The temperature of the part during the printing process was recorded in two ways. An infrared camera, a Teledyne FLIR (Wilsonville, OR, USA) a325sc was positioned so that it could observe the printed specimens horizontally. Additionally, thermocouples were introduced into the print by placing them on top of a printed line and holding them in place while the next layer was deposited on top and solidified, fixing the thermocouple in place. The measurements yielded a surface and core temperature for the printed lines and were used subsequently for the analysis of assumed surface-temperature-based bonding between layers and core-temperature-based crystallization of the polymer by replicating the measured thermal history.

#### 2.2.2. Sample Geometry and Parameter Settings

Sample geometry was chosen as cubes with 300 mm edge length and corners rounded to 50 mm radii in the printing plane, printed in a spiral mode. This yielded 200 mm by 300 mm flat plates on each side for sample preparation and allowed for the extrusion to be under almost stationary conditions. Coupons for subsequent testing were then milled from the plane faces, an approach which has already been validated on a smaller scale to yield more repeatable results than direct printing of the coupons [16].

For this, the cubes were separated into the four side planes. One was used for coupons for analysis of fiber orientation and two were used for coupons for mechanical testing by 3-point bending. The coupons for mechanical testing were cut on one along X and the other along Z. This allowed for testing of fiber-dominated behavior along X, and matrix-dominated behavior along Z. The specimens for the analysis of fiber alignment and porosity were cut so the XY and YZ planes were visible with fiber orientation determined in XY and porosity in YZ. The remaining plate had the thermocouples inside and was used for DSC analysis samples. Crystallization was then reproduced with the material and approximate thermal history of the respective cube.

The experimental set was based on parameter variation in line width, layer height, layer time, and extrusion temperature using a D-optimal design of experiments in Modde 13. The set consisted of 30 experiments including three center point repetitions. The observed responses and method of measurement were as follows in Table 1.

The factor ranges were as follows in Table 2.

#### 2.2.3. Machining of Coupons and Polishing of Cross-Sections

Coupons for mechanical testing were machined from the plates by first machining both sides of the plate flat to remove layer lines and then cutting the specimens by contour milling. The resulting coupons were smooth on all sides to avoid notch effects of the layers influencing mechanical characterization and provide homogeneous specimen shape throughout extrusion width and height settings. Flood cooling was used to minimize the temperature increase during milling, which could potentially weld the layers and affect the measured layer bonding. Samples were dried and then conditioned at 23 °C and 50% humidity for at least 48 h and kept in the climate chamber until their testing.

For cross-section microscopy, samples were embedded in transparent epoxy resin for stabilization prior to polishing. Polishing was conducted in steps on a water-lubricated disc grinder using silicon carbide sandpaper in grit steps of 180, 320, 800, 1200, 2400, and 4000 to achieve scratch-free surfaces for analysis. The cross-sections were recorded on an Olympus BX41M incident light optical microscope (Tokyo, Japan) with a 10× magnification and automatic multiple image alignment to record the entire extrusion line width in one image. Five specimens were imaged per parameter set. Analysis was conducted with a greyscale analyzing program previously used for the study of smaller specimens from fused filament fabrication [17].

### 2.3. Material Characterization

#### 2.3.1. DSC Measurement for Recreating Thermal History

DSC measurements were performed to accurately recreate the LFAM process’s thermal history. The DSC measurements were performed to investigate the occurrence of crystallization during the cooling cycle and determine if it was suppressed due to the fast cooling rates present in the additive manufacturing process.

The thermocouples introduced during printing provided time-continuous temperature data that allowed the replication of the three distinct cooling phases experienced by the material during printing.

**Figure 2:**
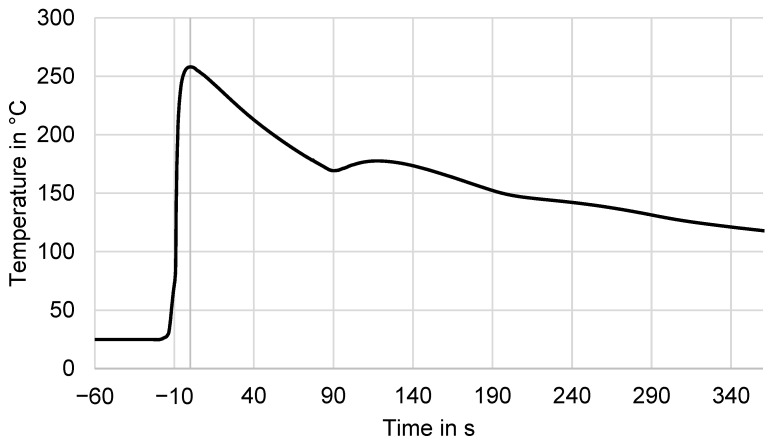
Exemplary cooling curve as recorded by thermocouple during experiment #9. The time zero point is set at the maximum recorded temperature at the deposition.

The three cooling phases consisted of the following:

1. Quench-Cooling: After extrusion, the fresh molten material immediately comes into contact with the print surface, leading to rapid quench-cooling. During this phase, the material was rapidly cooled to an elevated temperature slightly below the extrusion temperature. In Figure 2, this corresponds to the drop in the extrusion temperature of 350 °C to the maximum recorded temperature of 260 °C. This occurs within the 15 s from the first onset temperature increase at −15s and corresponds to a cooling rate of 360 K/min in this example.
2. Fast Cooling: Once the layer was deposited and free on three sides, fast cooling occurred to a second, lower temperature at time 0 to 90 s in Figure 2 to 170 °C. Cooling rates during this phase were between 60 and 120 K/min, in the example shown, at a relatively low rate of 60 K/min compared to the other experiments.
3. Slow Cooling: After the next layer was deposited, after a short increase in temperature, the cooling rate of the material, now only free on its sides, was reduced. This slower cooling phase could provide sufficient time for the material to achieve crystallization, if transition to the slow phase occurred at a high enough temperature level. Cooling rates in this phase were lower than 25 K/min, in the example, from 120 s onwards at on average 15 K/min until 360 s.

To recreate the three observed cooling phases, a Perkin Elmer (Shelton, CT, USA) DSC8500 was used. This DSC enabled cooling rates of up to 750 K/min, providing the capability to recreate the rapid cooling rates and measure the effect on material crystallization.

The cooling profiles were set to begin after a five-minute isothermal step at the extrusion temperature with a so-called “ballistic cooling” step in the instrument settings, which is uncontrolled cooling at the maximum achievable rate of the instrument to the first target temperature. From there, constant cooling occurred at the average rate of phase 2 to the temperature at which the next layer was deposited, determined by the lowest temperature measured by the thermocouple before the spike in temperature from the deposition. The last cooling step was conducted with the cooling rate determined by the pre-deposition temperature and the temperature recorded three minutes later. The sample was cooled at this rate until 120 °C, which is below the glass transition temperature. At that point, no more crystallization was expected, and it was held there in a two-minute isothermal step, allowing the sample and instrument to settle, before beginning a heating cycle at a constant 20 K/min to the extrusion temperature.

During cooling, the temperatures of onset, peak, and end of crystallization were observed. During heating, the melt enthalpy of crystals formed was determined. As the DSC is only calibrated for one heating rate, the temperatures determined during the cooling cycles may be less accurate than the values specified during heating. However, as crystallization generally occurred during the last stage of cooling, which was at rates lower than 25 K/min, and the instrument was calibrated for the 20 K/min of the heating cycle, the expected error is smaller than 1 K, which was accepted.

Crystallization of the material is important as it both hinders bonding of the layers if it occurs too early in processing and improves the material’s properties in the finished print if completed [18].

#### 2.3.2. Fiber Alignment

The alignment of fibers inside the printed lines through the squeeze flow underneath the nozzle has a strong effect on the mechanical properties [19,20,21]. Accurate assessment of the fiber orientation within the prints is crucial for understanding the resulting mechanical properties. A proven precise method for determining the fiber orientation is computed tomography; however, to achieve a resolution high enough to identify the individual fibers, specimens must be small and substantial amounts of data are generated. This makes it unpractical for the screening of sets with a high number of parameters and large specimens, as in this study. For this reason, for this study, we attempted to use image analysis of cross-section micrographs with the help of a previously developed Python script. The core assumption is that fibers are mostly oriented in the XY plane and cross-sections can be made parallel. This had previously proven accurate for fused filament fabrication printed specimens with low layer heights [17]. However, in this study, it became evident that the approach is less effective for LFAM, as the significantly higher layer heights result in more out-of-plane fiber orientation. With regions within the extruded line showing significantly different fiber alignment, highly aligned around the edges of the line and highly randomized in the core, the resulting distribution of the measurements is strongly affected by the position of the cross-section inside the line. This resulted in a very high variance in measurement results of repetitions on the same specimen and would only have been possible to remedy with a significant additional effort to ensure precise positioning of the cross-section, defeating the purpose of being able to analyze a large number of samples quickly. For interested readers, the results are still included in the tables in Appendix A but will not be included in the article. The measurement is performed by creating a histogram of all fiber orientations, which follows a normal distribution, and then determining the standard deviation of the distribution, effectively measuring the fiber misalignment rather than alignment.

#### 2.3.3. Mechanical Testing

Mechanical testing was performed by 3-point bending on a universal test machine, a UPM250 by Hegewald&Peschke (Neu-Ulm, Germany). The loads were measured using a 5 kN load cell, and deflection was measured using a DK830SLR extensometer by Magnescale (Tokyo, Japan). Conditioned specimens were removed from the climate chamber as a set and immediately tested. Six specimens were evaluated per orientation for each parameter set.

#### 2.3.4. Data Analysis

**Figure 3:**
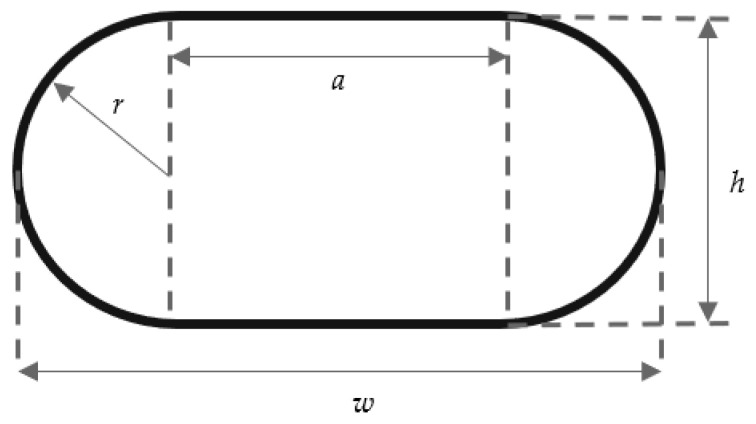
Approximated extrusion line cross-section as stadium shape.

In addition to the original four factors, extrusion width, extrusion height, extrusion temperature, and layer time, two calculated metrics were formed that consider the interactions of these factors. For both of these, the cross-section area of the extrusion line is used and approximated as a stadium or discorectangle shape, as shown in Figure 3, with the layer height *w* corresponding to the radius and the extrusion line width *w* to the line width, which is the length of the straight sides *a* and twice the radius. The extrusion line cross-section area $A_{EL}$ can then be calculated as:

$$A_{EL}=\pi \times {\left(\frac{h}{2}\right)}^{2}+h\times (w-h)$$

With this approximation, the following metrics can be calculated:

Enthalpy Deposition Rate $\dot{H}$: As the combination of extrusion temperature and material output, this metric is the enthalpy of the material deposition rate. It indicates the power at which thermal energy is introduced into the part. Output is calculated from the line cross-section, simplified as a rectangle of extrusion width by layer height, with Tool Center Point (TCP) speed indirectly set in the experiments by the layer time.

$$\dot{H}=\left(c\times \left(T_{Ex}-T_{A}\right)+H_{Crys_{Max}}\right)/\rho \times (A\times v_{TCP})$$

with specific heat *c*, extrusion temperature $T_{Ex}$, ambient temperature $T_{A}$, maximum crystallization enthalpy $H_{Crys\_Max}$, density *ρ*, and Tool Center Point Speed $v_{TCP}$.

This assumes that cooling of the part and the enthalpy deposition rate must form an equilibrium and the layer temperature will be related to this aggregate.

Shear Rate $\dot{\gamma }$: Pibulchinda et al. [19] have previously proposed using Jeffery’s equation [22] using empirical modifications to calculate the shear rate as a metric to investigate how shear and flow affect fiber orientation. This approach was also mentioned in previous publications [23,24,25,26,27,28,29]. The equation is modified from the formulation by Pibulchinda et al. using the extrudate and bed velocity, or in this case, nozzle velocity, by using the relation of nozzle orifice area $A_{N}$ to extrusion line cross-section $A_{EL}$. The extrudate velocity is calculated by assuming that the volume flow through the nozzle must be equal to that flowing into the extrusion line, with the ratio of flow velocities then equal to the inverse ratio of areas. By simplifying the line cross-section in the same manner as before, the shear rate can then be calculated from the factors of the experiment using the TCP speed of the machine, which depends on the set layer time, as follows:

$$\dot{\gamma }=\frac{v_{TCP}}{h}\times \left(1-\frac{A_{EL}}{A_{N}}\right)$$

The nozzle orifice area is assumed to be a circle with nozzle diameter $D_{Nozzle}$ and $A_{N}=\pi +(\frac{D_{Nozzle}}{2})$.

The effect of process responses is visualized by linking these metrics to mechanical properties as desired part responses, under the assumption that the enthalpy deposition rate will affect the properties dominated by welding processes between layers and shear rate the properties dominated by fiber orientation in the squeeze flow under the nozzle. This aims to improve the understanding of the material responses by linking them to process conditions resulting from the process parameters, which only indirectly affect the material responses.

## 3. Results and Discussion

A complete table of results for the experiments is included in Appendix A. The key results are summarized in graphs and interpreted in the following sections.

### 3.1. Thermal History and Crystallization

**Figure 4:**
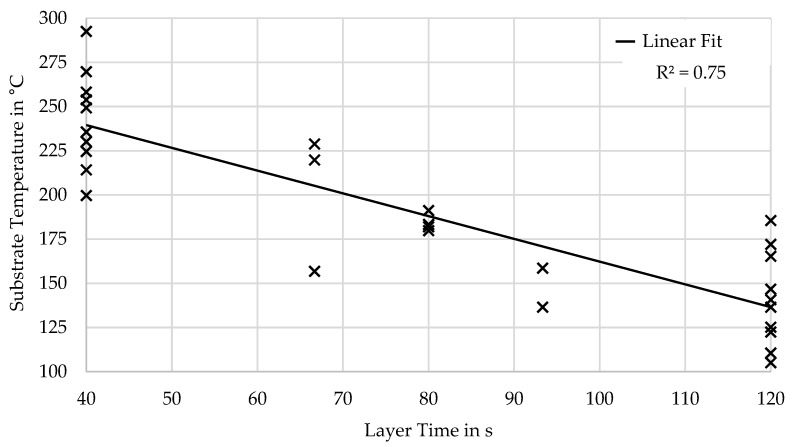
Correlation of substrate temperature over layer time.

The basic parameters showed no correlation to only a very weak correlation and high variance between the resulting layer temperatures during processing. Only the layer time was found to have a strong negative correlation with the substrate temperature. However, the substrate temperature varied by almost 100 °C for identical layer times, depending on the combination with the other parameters of extrusion height, extrusion width, and extrusion temperature, as can be seen in Figure 4.

**Figure 5:**
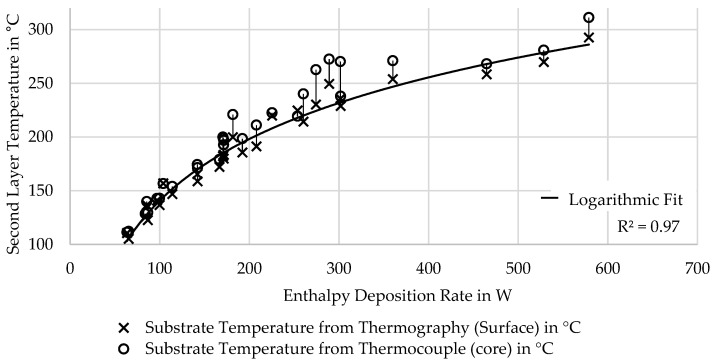
Measured substrate temperature by thermography and overprinted thermocouple, showing higher internal temperature in crystallization window between 180° and 260 °C.

The previously introduced enthalpy deposition rate showed much higher correlation with the resulting layer temperature just before deposition of the next layer, as can be seen in Figure 5. A comparison of temperature measurements during printing by thermography and introduced thermocouples is included in the same figure. As expected, the thermocouple readings show slightly higher temperature than the thermography, since they measure the inside of the deposition rather than the free surface. The difference between readings is demonstrated by the one-side error bars in Figure 5, with the marker indicating the reading of the IR camera and the end of the error bar the reading of the thermocouple. This difference is most prominent in the range between about 180 and 260 °C, which is close to the crystallization window of the material of about 180 to 245 °C. While the difference indicates that the temperature gradient within a single layer is small, mostly less than 10 °C, outside of the crystallization window, it can reach up to 35 °C when the layer is completed just as the crystallization reaches its peak.

Substrate surface temperature correlates with the enthalpy deposition rate, and experiments with very different process parameters achieve similar layer temperature if the aggregate is similar, as can be seen in the pairwise examples of Table 3.

**Figure 6:**
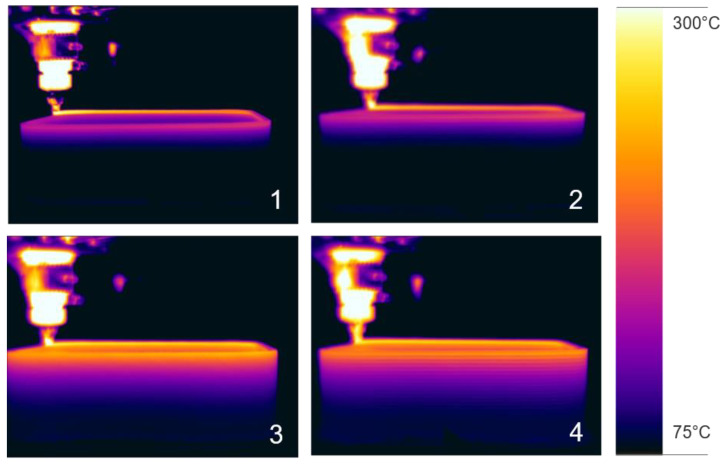
IR images of examples of Table 1, numbers 1–4 indicating the corresponding row of Table 3.

**Figure 7:**
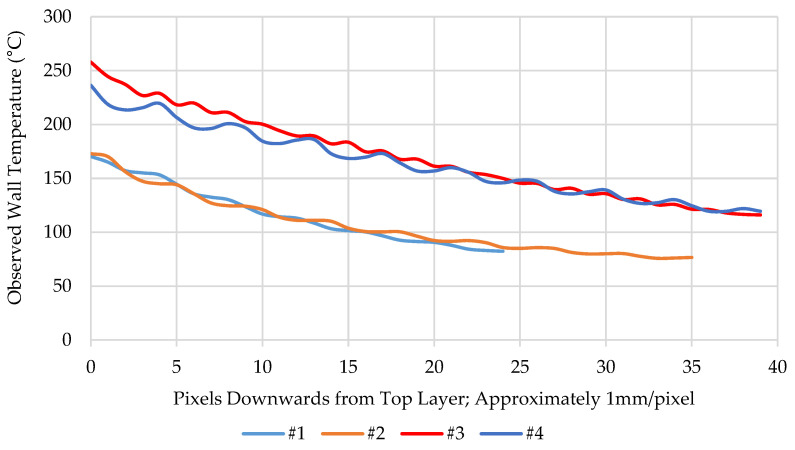
Temperature distribution in images of Figure 6 from top layer down along Z.

Figure 6 shows the side IR image of the prints, showing that for the observed temperature, it is similar not just for the last layer, but also for the material below, independent of the extrusion height, width, and temperature, as long as the enthalpy deposition rate is similar. Higher enthalpy deposition rate leads to a higher substrate temperature at contact with the deposition but also shifts the thermal profile along Z to a higher level, as can be seen in Figure 7. But while the spatial temperature distribution is very similar, due to the different build up rates, the cooling rates can be significantly different.

The difference of the readings of the surface by IR camera and internal thermocouple, as shown in Figure 5, increases when transitioning below about 260 °C, at which the internal thermocouple reading seems to plateau in the 300 to 400 W range, before achieving higher temperatures at higher enthalpy deposition rates. This matches the measured onset of crystallization during DSC. This could explain the relative increase in temperature of the thermocouple reading inside the material against the IR reading of the surface.

**Figure 8:**
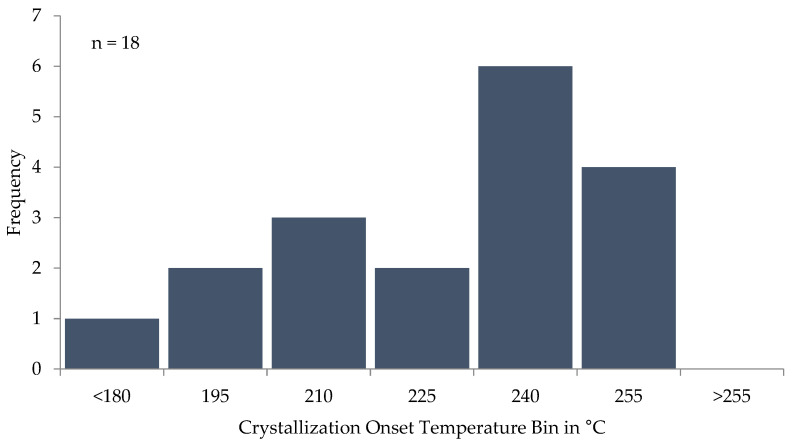
Crystallization onset histogram in DSC measurements recreating experiment cooling rates. Of the experiments, 18 showed crystallization, and for the remainder, it was fully suppressed.

**Figure 9:**
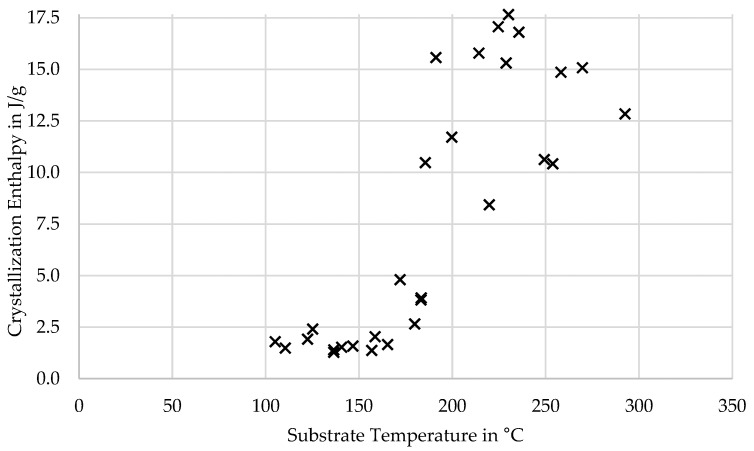
Crystallization enthalpy over substrate temperature.

The DSC measurement also showed that the achieved crystallinity was highest for experiments in which the consecutive layers were deposited when the layer had cooled to temperatures slightly above the window of crystallization onset. A higher layer temperature showed lower crystallinity, as shown by the histogram in Figure 8. This is likely due to the two-staged nature of crystal growth, with nucleation preceding crystal growth. Supercooling the melt causes a higher nucleation rate but decreases crystal growth rate. An optimum for crystallization seems to exist at which a supercooling to the crystallization onset temperature causes a high nucleation rate and seeds crystals which can grow in the subsequent slower cooling phase. Higher layer temperature may either result in less nuclei being formed or nuclei being dissolved by the temperature increase caused by the deposition of the next layer, as can be seen in Figure 9. Another plausible explanation could be the increased formation of a higher enthalpy crystal phase, as indicated by the multiple melting peaks [30,31,32]. If the layer had cooled below ~200 °C, the crystallization was almost suppressed.

### 3.2. Fiber Misalignment

**Figure 10:**
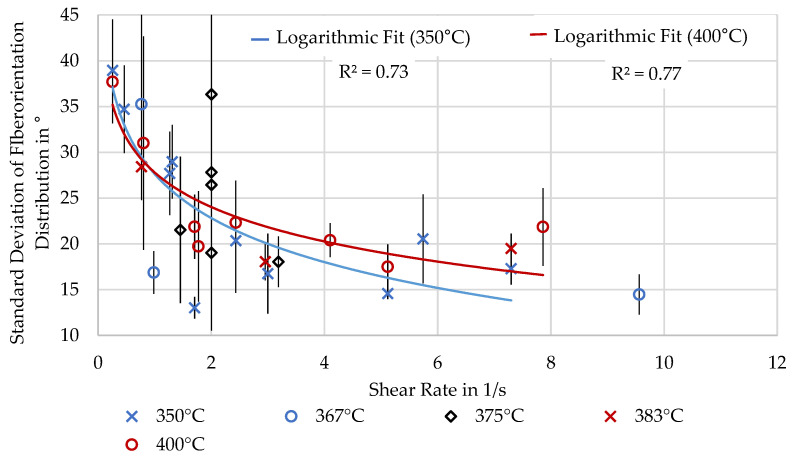
Fiber misalignment over shear rate.

Determining the fiber orientation through micrographs proved susceptible to measurement errors. Therefore, the results should be seen as indicative values and are only included for completeness. A trend to more aligned fibers with shear rate was noticeable and agrees with the determined fiber-dominated mechanical properties in X. For completeness, this trend is shown in Figure 10 over the shear rate.

Overall, the experiments agree with the expectation that a higher shear rate leads to lower fiber misalignment. However, because of the flawed measurement technique, no conclusion should be drawn due to the high variance of measurement results. Logarithmic trendlines have been added for the highest and lowest extrusion temperature experiments, respectively, 400 and 350 °C, which have a difference in viscosity. The trend for lower temperatures points towards better alignment at high temperatures, which is plausible, as the higher viscosity would lead to higher stress and larger aligned edge regions in the extrusion line.

### 3.3. Mechanical Properties

The measured thermal history and microstructure of fiber alignment inside the lines during processing were expected to have a strong effect on the mechanical properties of the resulting part, which three-point bending tests could confirm. The results showed that the intermediate step of thermal history has a clear correlation with the resulting matrix-dominated mechanical properties. In contrast, the impact of the material flow on fiber-dominated properties along the extrusion lines could only show a tendency without a clear correlation.

#### 3.3.1. Matrix and Thermal History Dominated in Z

**Figure 11:**
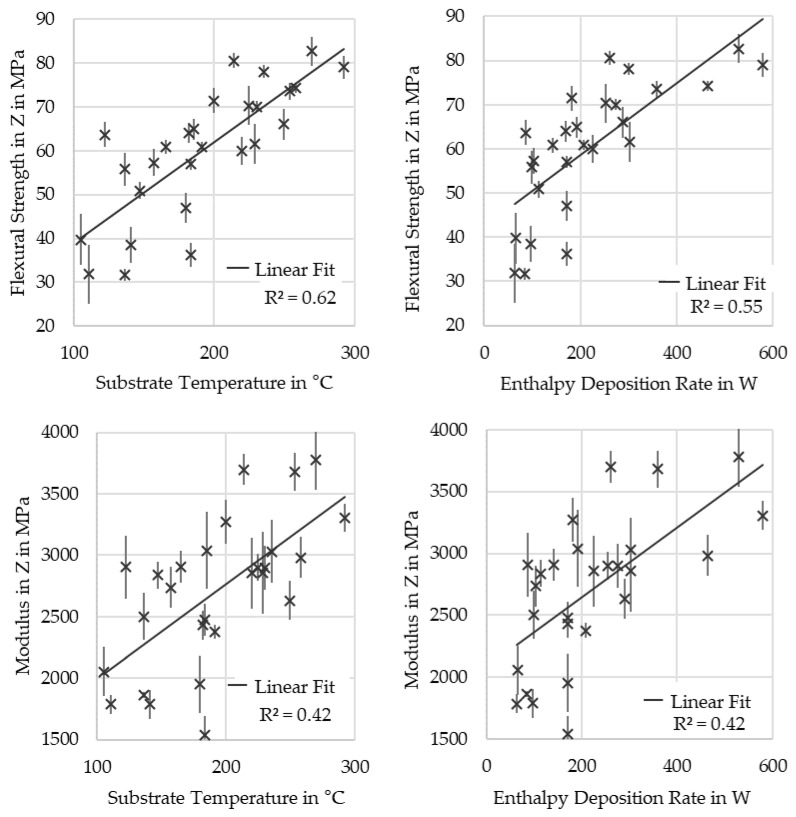
Three-point bending strength and modulus in Z over substrate temperature and enthalpy deposition rate.

The matrix dominates the mechanical properties of the part perpendicular to the layer plane. Strength is mostly determined by the bonding of the layers through welding by chain diffusion. This is a strongly temperature-driven process, and an increase in substrate layer temperature at the time of deposition leads to an increase in strength, as shown in Figure 11. As the substrate temperature depends strongly on the enthalpy deposition rate, as previously described, and substrate temperature influences strength and stiffness; a correlation between deposition power and mechanical properties was also found, as seen in the figures. However, as fusion bonding is also a time-dependent process, cooling rates from the substrate temperatures will also have an effect that is not captured by this metric.

A similar dependence was found for the determined modulus of the specimens. As fibers align mostly in the layer plane, this increase is expected to be due to a higher crystallinity of the matrix and a more difficult crack propagation along layer interfaces [33].

**Figure 12:**
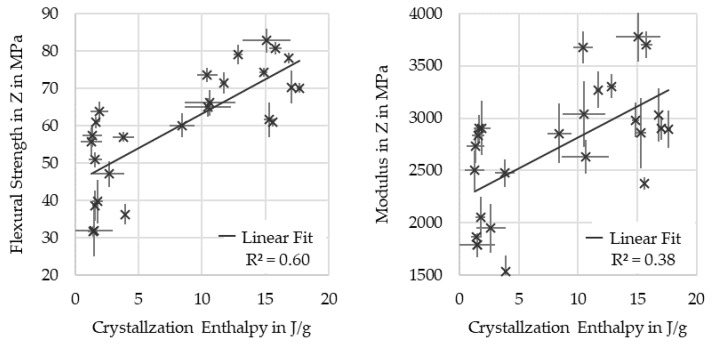
Strength and stiffness over crystallization enthalpy.

Figure 12 shows that samples with high crystallinity have high mechanical properties; however, the correlation is weak. Results above 70 MPa strength and 3000 MPa stiffness occurred only at high crystallinity. However, the presence of results with lower mechanical properties at high crystallinity indicate that other influencing factors are present.

Overall, it can be concluded that higher deposition rates leading to higher processing temperatures improve the matrix-dominated material properties. However, there is a natural limitation to this. One of the experiments, with an extrusion temperature of 400 °C, line width of 12 mm, layer height of 4 mm, and layer time of 40 s, resulting in a deposition power of 579 W and a substrate layer temperature of 311 °C, was on the limit of collapse and substrate layers were pushed aside during printing. Testing was only possible as line width was high enough to machine deep enough into an uncompromised layer stack.

#### 3.3.2. Fiber Alignment and Material Flow Dominated in X

**Figure 13:**
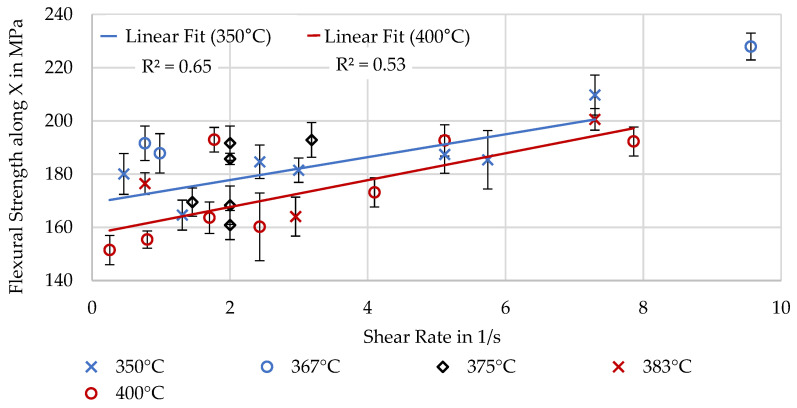
Flexural strength determined by 3-point bending along X.

**Figure 14:**
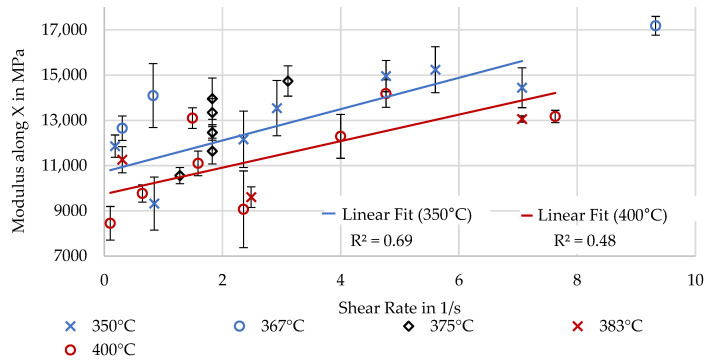
Modulus determined through 3-point bending along X.

To investigate the effect of extrusion parameters on mechanical properties affected by fiber alignment, the shear rate is used. As the measurement of fiber alignment was inaccurate and unreliable, the mechanical properties were not correlated to these measurements. Figure 13 and Figure 14 illustrate that a correlation between the shear rate and the mechanical properties exists, with high shear rates leading to high strength and stiffness. This is assumed to be due to a higher fiber alignment. Linear trendlines have been added for the experiments at 350 and 400 °C again to give an indication of the effect of lower viscosity. The trendline for the cooler and, thereby, more viscous material shows higher material strength and stiffness, possibly due to more fibers being aligned through thicker edge regions with high alignment. These experimental results agree with the simulated results of Pibulchinda et al. [19].

**Figure 15:**
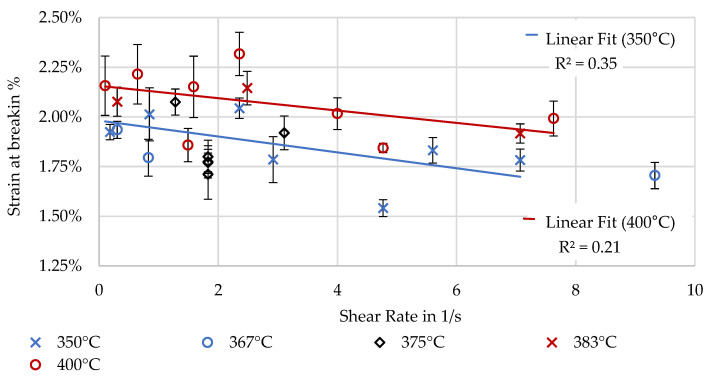
Strain at break determined through 3-point bending along X.

Strain at break is low for all specimens with only weak correlation to the shear rate, shown in Figure 15. A higher strain at break at higher temperatures is visible for higher extrusion temperatures.

### 3.4. Effects of Extrusion Parameters

The results show indirect correlations between the settable extrusion parameters and the resulting material properties, with their interactions having a more direct impact, as seen from the calculated metrics deposition power and shear rate. While the shear rate affects mostly fiber-dominated properties of the material, the deposition affects mostly the matrix-dominated properties.

Fiber misalignment was expected to strongly affect the mechanical properties of the material, but it could not be accurately measured using only microscopy. However, a high degree of fiber misalignment leads to lower stiffness and strength along the extrusion lines. High values of shear rate were shown to improve these, suggesting that it also helps in aligning the fibers inside the extrusion lines [24,34,35]. This trend fits well with the mean values of the observed fiber misalignment. Large layer heights resulted in more inaccurate measurements of the fiber misalignment and a larger portion of the line core having high out-of-plane fiber misalignment. This behavior is similar to injection molding, where the fiber misalignment in the core increases [36,37]. A design guideline like a maximum wall thickness in injection molding for a maximum extrusion line height and possibly also for the ratio of width to height in MEX should be considered.

The thermal history of the part during printing has a strong influence on the matrix material, affecting the welding process between the layers, the crystallization, and warping of the part. This temperature strongly correlates with the deposition power of the process, with different extrusion settings resulting in similar thermal conditions when the aggregates are similar. Higher deposition powers lead to higher substrate temperatures during deposition. This also suggests that the common approach of using a set layer time is not valid if the layer height or line width is changed.

Perpendicular to the layer plane, the matrix dominates the mechanical properties. The strength developed during bonding of the layers through chain diffusion is a temperature-driven process. The substrate layer temperature during deposition is a key factor affecting the final part strength. Higher substrate temperatures strengthen bonding and improve mechanical performance [38,39].

Layer temperature and cooling rates are also critical for the crystallization behavior, and the experiments demonstrate that higher layer temperatures lead to higher crystallinity. However, there is an optimum temperature range for crystallization, and excessively high layer temperatures can result in lower degrees of crystallinity. Highly crystalline samples showed high stiffness compared to samples with low crystallinity.

AM materials with a slower crystallization kinetic, like the investigated PAEK material, show significantly enhanced strength between layers compared to materials designed for injection molding with faster crystallization kinetics [12,40]. For end-use applications, crystallization must be completed to ensure the desired material properties. Yet, the experiments have shown that for a wide range of layer temperatures, a high degree of crystallinity can be reached [41,42]. This would make post-processing annealing necessary. The transition to full suppression is fast when the material cools fast enough to affect the crystallinity developed.

## 4. Conclusions

This experimental study emphasizes the importance of extrusion parameters in LFAM to optimize the mechanical properties of printed parts [43]. The results provide insights for designing end-use parts with LFAM, particularly when using high-performance polymers with fiber reinforcement. As the investigated aggregates affecting the material properties are influenced by the same extrusion parameters, such as layer height, line width, and print speed, not all can be adjusted independently to achieve a desired outcome of material properties, and they need to be considered with their interactions.

The enthalpy deposition rate should always be maximized to ensure that the layers are bonded together; this results in high crystallinity and productivity. This can be achieved both through small lines at high print speeds or large lines and small print speeds; however, material degradation and part slumping set limits on how far this can be increased. The shear rate can be adjusted to achieve a desired fiber alignment, stiffness, and strength along X.

If the highest strength and stiffness are desired, the shear rate should be high by using small extrusion line dimensions and high print speed, ideally with extrusion of the same width as the nozzle orifice diameter to avoid material flow and fiber alignment in the Y direction, in the print plane but perpendicular to the movement direction. On the other hand, if a more isotropic behavior with more random fiber orientation is desired, shear rates should be lower, by using large lines printed at low speeds, ideally overexpanding the line to widths larger than the nozzle orifice diameter to achieve a squeeze flow under the nozzle with the resulting fiber misalignment from the movement direction.

The shear rate depends only on the squeeze flow under the nozzle and can be predicted with little effort to design the process according to the desired material morphology. Layer temperature depends on the balance of introduced energy through the enthalpy deposition rate and the dissipated heat from the part that is dependent on the geometry and ambient conditions and therefore much more difficult to predict. It would be beneficial to develop a system to monitor and adjust this in real time during the process. It is likely easier to control in a closed loop by adjusting cooling or introducing energy as needed, or if possible, without compromising the shear rate and affecting the properties along X, by adjusting the machine speed.

## Figures and Tables

**Table 1:** Responses measured in the experimental design.

Response	Measurement Method	Unit
Material core temperature at deposition (Temperature the material is quench-cooled to)	Thermocouple overprinted	°C
Material core temperature at second layer (Target temperature for fast cooling in DSC cycles)	Thermocouple overprinted	°C
Cooling rate (high): Topmost layer	Thermocouple overprinted	K/min
Cooling rate (low): After coverage with additional layers	Thermocouple overprinted	K/min
Layer contact temperature: surface just before nozzle	IR Camera	°C
Fiber misalignment	Image analysis on cross-section micrographs in XY plane	Standard deviation of orientation distribution in °
Bending strength along extrusion lines (fiber-dominated)	3-point bending	MPa
Bending strength across layers (matrix-dominated)	3-point bending	MPa
Elastic modulus along extrusion lines (fiber-dominated)	3-point bending	MPa
Elastic modulus across layers (matrix-dominated)	3-point bending	MPa
Strain at break along extrusion lines (fiber-dominated)	3-point bending	%
Strain at break across layers (matrix-dominated)	3-point bending	%
Crystallization onset (temperature and time)	DSC of feedstock	°C/s
Crystallization peak (temperature and time)	DSC of feedstock	°C/s
Crystallization enthalpy	DSC of feedstock	J/g
Relative crystallinity	DSC of feedstock	%

**Table 2:** Factor ranges set in the experimental design.

Parameter	Lower Limit	Upper Limit
Line width	8 mm (100% of nozzle diameter)	12 mm (150% of nozzle diameter)
Layer height	2 mm	4 mm
Extrusion temperature	350 °C	400 °C
Layer time	40 s	120 s

**Table 3:** Pairwise comparison of experiments with similar deposition powers but different extrusion parameters.

Number in Figure 6	Extrusion Temperature in °C	Extrusion Width in mm	Layer Height in mm	Layer Time in s	Enthalpy Deposition Rate in W	Surface Temperature in °C
1	350	8	4	93	142.1	170.3
2	400	9	4	120	141.9	172
3	400	12	2	40	301.6	257.7
4	350	12	4	67	301.8	236.9

## Data Availability

The raw data supporting the conclusions of this article will be made available by the authors upon request.

## Appendix A

**Table A1:** Full list of factor settings.

	Set Factors				Calculated Metrics		
Number	Temperature in °C	Extrusion Width in mm	Extrusion Height in mm	Layer Time in s	TCP Speed in mm/s	Shear Rate in 1/s	Enthalpy Deposition Rate in W
1	350	12	2	40	27.05	7.298	260.30
2	400	12	2	40	27.05	7.298	301.58
3	400	12	4	40	27.05	0.767	578.87
4	350	12	2	120	9.02	2.433	86.67
5	400	12	2	120	9.02	2.433	99.79
6	350	12	4	120	9.02	0.256	166.59
7	400	12	4	120	9.02	0.256	192.17
8	350	8	2	67	16.43	5.742	103.87
9	350	8	4	93	11.73	1.266	142.12
10	350	8	3	40	27.37	5.117	253.65
11	350	8	3	120	9.12	1.705	83.87
12	350	12	4	67	16.25	0.461	301.84
13	350	9	2	120	9.10	2.998	65.25
14	350	11	4	40	27.13	1.309	464.80
15	400	8	2	93	11.73	4.099	85.49
16	400	8	4	67	16.43	1.774	225.31
17	400	8	3	40	27.37	5.117	289.09
18	400	8	3	120	9.12	1.705	96.91
19	400	11	2	40	27.13	7.861	274.31
20	400	9	4	120	9.10	0.801	141.90
21	366.7	8	2	40	27.37	9.561	181.54
22	383.3	8	2	120	9.12	3.185	63.45
23	383.3	8	4	40	27.37	2.953	360.03
24	366.7	8	4	120	9.12	0.984	114.01
25	366.7	12	4	40	27.05	0.767	528.51
26	375	12	3	80	13.53	1.454	207.82
27	375	10	3	80	13.60	2.002	171.48
28	375	10	3	80	13.60	2.002	171.30
29	375	10	3	80	13.60	2.002	170.91
30	375	10	3	80	13.60	2.002	170.58

**Table A2:** Responses of the experiments, part 1 of 2.

	Responses Part 1							
Number	Material Core Temperature at Deposition in °C	Material Core Temperature at Second Layer in °C	Cooling Rate (High): Topmost Layer in °C/min	Cooling Rate (Low): After Coverage with Additional Layers in °C/min	Layer Contact Temperature: Surface Just before Nozzle in °C	Fiber Misalignment in ° with Standard Dev.	Bending Strength along Extrusion Lines (Fiber-Dominated) in MPa with Standard Dev.	Bending Strength across Layers (Matrix-Dominated) in MPa with Standard Dev.
1	275.60	240.00	60	13	214.20	17.30 ± 1.75	209.71 ± 7.52	80.60 ± 1.66
2	315.86	270.12	92	13	235.60	19.50 ± 1.61	200.57 ± 4.07	78.08± 1.41
3	357.32	311.10	67	18	292.50	28.43 ± 3.63	176.42 ± 4.06	79.03 ± 2.70
4	264.95	129.56	69	−1	122.40	20.35 ± 5.71	184.63 ± 6.31	63.68 ± 2.84
5	289.64	142.84	77	−2	136.50	22.33 ± 4.57	160.17 ± 12.70	55.82 ± 3.74
6	292.78	179.00	56	6	172.00	38.95 ± 5.57	NA	NA
7	349.14	198.38	73	6	185.50	37.70 ± 4.53	151.44 ± 5.53	64.88 ± 2.45
8	248.70	156.60	90	7	156.80	20.55 ± 4.86	185.38 ± 11.00	57.29 ± 3.03
9	260.38	171.33	61	11	158.60	27.70 ± 4.55	NA	NA
10	277.50	219.25	104	16	224.60	14.58 ± 0.62	187.37 ± 7.06	70.31 ± 4.41
11	266.70	128.48	70	3	125.20	13.03 ± 1.19	NA	NA
12	282.10	237.85	46	13	228.80	34.7 ± 4.79	180.03 ± 7.66	61.54 ± 4.57
13	252.99	111.97	73	−2	105.10	16.75 ± 4.37	181.46 ± 4.58	39.74 ± 5.79
14	308.83	268.01	48	17	258.20	28.98 ± 4.03	164.59 ± 5.63	74.27 ± 1.14
15	325.98	139.81	123	2	136.50	20.40 ± 1.86	173.13 ± 5.52	31.68 ± 1.36
16	292.26	222.46	65	17	219.80	19.73 ± 6.03	192.92 ± 4.62	60.03 ± 3.22
17	343.44	272.37	105	22	249.30	17.50 ± 2.44	192.66 ± 5.89	66.06 ± 3.55
18	328.86	142.65	99	1	140.70	21.88 ± 3.51	163.62 ± 5.90	38.50 ± 4.03
19	314.73	262.62	95	14	230.10	21.85 ± 4.25	192.25 ± 5.45	70.06 ± 1.29
20	344.27	174.25	84	5	165.30	31.00 ± 11.67	155.40 ± 3.24	60.83 ± 1.63
21	276.93	220.79	94	16	199.70	14.48 ± 2.19	227.92 ± 5.04	71.45 ± 2.87
22	265.19	111.23	80	−2	110.50	18.05 ± 2.78	192.85 ± 6.55	31.85 ± 6.74
23	307.53	270.98	82	21	253.80	18.07 ± 2.06	164.03 ± 7.32	73.54 ± 1.96
24	297.00	153.83	80	3	146.70	16.88 ± 2.33	187.80 ± 7.39	50.85 ± 1.94
25	302.90	280.78	44	18	269.70	35.25 ± 9.88	191.57 ± 6.48	82.75 ± 3.28
26	305.55	210.99	76	8	191.20	21.53 ± 8.01	169.49 ± 5.31	60.82 ± 1.13
27	277.01	196.72	67	10	183.30	36.33 ± 8.77	160.87 ± 5.48	36.25 ± 2.73
28	289.63	192.61	76	9	179.80	27.83 ± 6.94	168.29 ± 7.26	47.06 ± 3.43
29	292.92	198.71	74	10	183.20	26.45 ± 8.59	185.73 ± 2.10	57.01 ± 1.29
30	296.36	199.83	79	10	182.00	19.03 ± 8.49	191.63 ± 6.43	64.00 ± 2.31

**Table A3:** Responses of the experiments, part 2 of 2.

	Responses Part 2							
Number	Elastic Modulus along Extrusion Lines (Fiber-Dominated) in MPa with Standard Dev.	Elastic Modulus across Layers (Matrix-Dominated) in MPa with Standard Dev.	Strain at Break along Extrusion Lines (Fiber-Dominated) in % with Standard Dev.	Strain at Break across Layers (Matrix-Dominated) in % with Standard Dev.	Crystallization Onset (Temperature and Time)	Crystallization Peak (Temperature and Time)	Crystallization Enthalpy in J/g	Relative Crystallinity
1	14,442.09 ± 882.07	3699.49 ± 128.40	1.78 ± 0.06	2.27 ± 0.06	232.36 °C/89.7 s	219.82 °C/146.72 s	15.79	89%
2	13,056.70 ± 139.78	3030.32 ± 258.25	1.92 ± 0.05	2.90 ± 0.11	240.13 °C/137.26 s	214.87 °C/246.4 s	16.80	95%
3	11,260.26 ± 580.24	3304.45 ± 116.92	2.08 ± 0.07	2.62 ± 0.08	242.03 °C/305.84 s	220 °C/391.5 s	12.84	73%
4	12,162.61 ± 1246.39	2905.32 ± 257.26	2.04 ± 0.05	2.40 ± 0.08	NA	NA	1.92	11%
5	9066.43 ± 1696.87	2502.00 ± 191.26	2.32 ± 0.11	2.62 ± 0.18	NA	NA	1.28	7%
6	NA	NA	NA	NA	NA	NA	4.81	27%
7	8449.26 ± 742.96	3039.19 ± 311.83	2.16 ± 0.15	2.24 ± 0.13	195.01 °C/128.846 s	185.15 °C/199.04 s	10.48	59%
8	15,237.24 ± 1015.82	2735.67 ± 166.47	1.83 ± 0.06	2.47 ± 0.17	NA	NA	1.37	8%
9	NA	NA	NA	NA	163.31 °C/216.3 s	50.29 °C/284.04 s	2.04	12%
10	14,957.735 ± 690.65	2898.58 ± 110.57	1.54 ± 0.04	2.71 ± 0.20	231.95 °C/78.54 s	217.92 °C/131.22 s	17.07	97%
11	NA	NA	NA	NA	219 °C/82.17 s	124.09 °C/100.17 s	2.41	14%
12	11,860.29 ± 493.62	2858.21 ± 334.94	1.92 ± 0.04	1.82 ± 0.11	228.43 °C/111.04 s	214.28 °C/174.98 s	15.31	87%
13	13,539.99 ± 1219.94	2054.35 ± 195.74	1.79 ± 0.12	2.06 ± 0.36	NA	NA	1.80	10%
14	9321.80 ± 1171.16	2982.68 ± 167.64	2.01 ± 0.13	2.55 ± 0.11	243.21 °C/131.36 s	225.57 °C/189.6 s	14.86	84%
15	12,292.56 ± 969.49	1864.83 ± 24.20	2.02 ± 0.08	2.34 ± 0.20	NA	NA	1.40	8%
16	13,099.85 ± 458.59	2854.93 ± 287.18	1.86 ± 0.08	2.29 ± 0.14	214.05 °C/161.52 s	198.14 °C/213 s	8.44	48%
17	14,182.86 ± 607.97	2632.01 ± 159.46	1.84 ± 0.02	2.73 ± 0.14	226.98 °C/160.54 s	221.62 °C/217.26 s	10.63	60%
18	11,098.65 ± 543.68	1787.53 ± 116.11	2.15 ± 0.15	2.71 ± 0.32	NA	NA	1.53	9%
19	13,170.27 ± 271.35	2896.77 ± 177.64	1.99 ± 0.09	2.46 ± 0.14	229.28 °C/137.44 s	204.3 °C/233.7 s	17.66	100%
20	9767.92 ± 381.98	2904.11 ± 129.96	2.21 ± 0.15	2.40 ± 0.11	NA	NA	1.66	9%
21	17,182.23 ± 412.13	3271.46 ± 176.69	1.70 ± 0.07	2.34 ± 0.18	208.51 °C/97.08 s	194.57 °C/147.66 s	11.71	66%
22	14,738.96 ± 668.09	1786.57 ± 78.67	1.92 ± 0.09	2.33 ± 0.77	NA	NA	1.49	8%
23	9605.70 ± 456.95	3679.72 ± 152.73	2.14 ± 0.08	2.31 ± 0.09	227 °C/126.87 s	199.80 °C/206.04 s	10.42	59%
24	14,095.51 ± 1410.61	2838.41 ± 110.80	1.79 ± 0.09	2.21 ± 0.12	NA	NA	1.59	9%
25	12,653.63 ± 537.95	3781.61 ± 245.08	1.93 ± 0.04	2.46 ± 0.09	241.25 °C/305.34 s	209.94 °C/399.58 s	15.08	85%
26	10,558.26 ± 359.14	2376.69 ± 60.07	2.07 ± 0.07	2.08 ± 0.08	201.84 °C/129.6 s	189.60 °C/208.77 s	15.58	88%
27	11,640.79 ± 566.11	1537.75 ± 151.19	1.80 ± 0.08	2.33 ± 0.31	180.61 °C/192.3 s	90.19 °C/233.04 s	3.92	22%
28	12,463.96 ± 340.84	1950.33 ± 234.21	1.71 ± 0.13	2.10 ± 0.14	NA	NA	2.66	15%
29	13,342.17 ± 619.77	2475.26 ± 130.41	1.77 ± 0.08	1.98 ± 0.10	192.54 °C/189.87 s	183.09 °C/235.02 s	3.82	22%
30	13,958.45 ± 915.31	2430.76 ± 117.29	1.77 ± 0.05	2.23 ± 0.08	NA	NA	0.00	0%
